# Variants in Iron Metabolism Genes Predict Higher Blood Lead Levels in Young Children

**DOI:** 10.1289/ehp.11233

**Published:** 2008-04-24

**Authors:** Marianne R. Hopkins, Adrienne S. Ettinger, Mauricio Hernández-Avila, Joel Schwartz, Martha María Téllez-Rojo, Héctor Lamadrid-Figueroa, David Bellinger, Howard Hu, Robert O. Wright

**Affiliations:** 1 Department of Environmental Health, Harvard School of Public Health, Boston, Massachusetts, USA; 2 Department of Medicine, Children’s Hospital Boston, Boston, Massachusetts, USA; 3 Channing Laboratory, Brigham and Women’s Hospital, Boston, Massachusetts, USA; 4 Department of Environmental Health Sciences, University of Michigan School of Public Health, Ann Arbor, Michigan, USA; 5 Ministry of Health, Mexico City, Mexico; 6 Division of Program Evaluation and Biostatistics, Center of Evaluation Research and Surveys, National Institute of Public Health, Cuernavaca, Morelos, Mexico; 7 Department of Neurology, Children’s Hospital Boston, Boston, Massachusetts, USA

**Keywords:** *C282Y*, children, *H63D*, hemochromatosis, iron, lead, *P570S*, polymorphism, transferrin

## Abstract

**Background:**

Given the association between iron deficiency and lead absorption, we hypothesized that variants in iron metabolism genes would predict higher blood lead levels in young children.

**Objective:**

We examined the association between common missense variants in the hemochromatosis (*HFE*) and transferrin (*TF*) genes and blood lead levels in 422 Mexican children.

**Methods:**

Archived umbilical cord blood samples were genotyped for *HFE* (*H63D* and *C282Y*) and *TF* (*P570S*) variants. Blood lead was measured at 24, 30, 36, 42, and 48 months of age. A total of 341 subjects had at least one follow-up blood lead level available and data available on covariates of interest for inclusion in the longitudinal analyses. We used random-effects models to examine the associations between genotype (*HFE*, *TF*, and combined *HFE* + *TF*) and repeated measures of blood lead, adjusting for maternal blood lead at delivery and child’s concurrent anemia status.

**Results:**

Of 422 children genotyped, 17.7, 3.3, and 18.9% carried the *HFE H63D*, *HFE C282Y*, and *TF P570S* variants, respectively. One percent of children carried both the *HFE C282Y* and *TF P570S* variants, and 3% of children carried both the *HFE H63D* and *TF P570S* variants. On average, carriers of either the *HFE* (β = 0.11, *p* = 0.04) or *TF* (β = 0.10, *p* = 0.08) variant had blood lead levels that were 11% and 10% higher, respectively, than wild-type subjects. In models examining the dose effect, subjects carrying both variants (β = 0.41, *p* = 0.006) had blood lead 50% higher than wild-type subjects and a significantly higher odds of having a blood lead level > 10 μg/dL (odds ratio = 18.3; 95% confidence interval, 1.9–177.1).

**Conclusions:**

Iron metabolism gene variants modify lead metabolism such that *HFE* variants are associated with increased blood lead levels in young children. The joint presence of variant alleles in the *HFE* and *TF* genes showed the greatest effect, suggesting a gene-by-gene-by-environment interaction.

Despite efforts to reduce lead in the environment by removing lead in gasoline and banning lead-based paint, an estimated 310,000 U.S. children 1–5 years of age have elevated blood lead levels [Centers for Disease Control and Prevention (CDC) 2005]. In developing countries, lead exposure is a concern because of continued use of lead-containing products and lack of regulations or enforcements policies (Meyer et al. 2003). Furthermore, research suggests that lead exerts its neurotoxic effects in children at blood levels lower than the current CDC action level of 10 μg/dL (Canfield et al. 2003; Jusko et al. 2008; Lanphear et al. 2005). There is growing interest in identifying host factors that increase the risk of elevated blood lead levels in children. Gene variants within metabolic pathways that influence lead absorption may be such a susceptibility factor and could place children at increased risk of lead poisoning even at low environmental levels (Onalaja and Claudio 2000).

Because of the well-described inverse relationship between iron stores and lead absorption (Kwong et al. 2004), iron metabolism genes are potential candidates to modify lead absorption and stores. Bradman et al. (2001) and Hammad et al. (1996) showed an inverse relationship between dietary iron and blood lead levels, and Wright et al. (1999, 2003) found an association between biomarkers of iron deficiency and elevated blood lead levels both cross-sectionally and longitudinally.

Iron absorption is regulated by iron stores and erythropoiesis (Bothwell et al. 1979) and is influenced by dietary iron intake and hypoxia (Morgan and Oates 2002). Hereditary hemochromatosis (HH) is an autosomal recessive disorder leading to excessive iron stores secondary to increased intestinal iron absorption (McLaren et al. 1991). Two predominant variants of the hemochromatosis (*HFE*) gene account for most cases: the *C282Y* and *H63D* variants, which are common in the U.S. population, with a prevalence of 7–17% and 10–32%, respectively (Bradley et al. 1998).

Transferrin forms a stable complex with the HFE protein to facilitate iron transfer (Feder et al. 1998; Lee et al. 1999). It has been suggested that the effects of *HFE* gene on iron absorption depend on the complex relationship between HFE and the transferrin receptor (TfR) (Morgan and Oates 2002). Feder et al. (1998) demonstrated that the *HFE H63D* variant altered transferrin binding, leading to a loss of HFE-repressor function for transferrin uptake, and thereby increasing iron transport into cells. A common missense variant of the transferrin gene (*TF*) is *P570S*, with a prevalence rate of about 15% in the general population (Lee et al. 1999).

Previous reports suggest that subjects with clinical hemochromatosis have higher (Barton et al. 1994) or equivalent (Åkesson et al. 2000) blood lead levels compared with normal controls. These studies were not population based, but used case–control designs comparing adult subjects with clinical disease with those without. In a population-based cohort, our group reported lower bone lead levels among elderly men carrying at least one copy of *H63D* or *C282Y* (Wright et al. 2004). The differences in the design, sex, and age of the different cohorts studied might account for the seemingly disparate findings, with older, male subjects with *HFE* variants being most likely to have high body iron stores and down-regulated lead absorption. In our 2004 report (Wright et al. 2004), we hypothesized that variants in iron metabolism genes might predict higher blood lead levels in young children over time because of their greater dietary iron needs and lower body iron stores, which would up-regulate iron and lead absorption differentially among *HFE* variant carriers and children with wild-type genotypes. In this study, we sought to determine whether genetic variants of iron metabolism (*HFE C282Y, HFE H63D, TF P570S*) were associated with blood lead levels in children.

## Materials and Methods

### Study population

Study participants were identified from among pregnant women receiving antenatal care between 1994 and 1995 at three hospitals in Mexico City that serve low- to middle-income populations. The women were approached before giving birth to participate in a randomized trial of calcium supplementation to lower blood lead levels over the course of lactation (Hernández-Avila et al. 2003). Infant development was also measured as part of this study, which was conducted as part of an established interinstitutional collaborative effort between researchers in the United States (Harvard University) and Mexico [National Institute of Public Health and American British Cowdray (ABC) Hospital]. The research protocol was approved by the Human Subjects Committees of the Harvard School of Public Health and the National Institute of Public Health and the participating hospitals in Mexico and has complied with all federal guidelines governing the use of human participants.

Data collection methods and exclusion criteria have been described in detail elsewhere (González-Cossío et al. 1997). Interviewers explained the study to and obtained written informed consent from eligible pregnant women who were willing to participate. Anthropometric data from the mother and newborn as well as umbilical cord and maternal venous blood samples were gathered within 12 hr of delivery. Information on estimated gestational age and characteristics of the birth and newborn period was extracted from the medical records. Baseline maternal information on reproductive and health status, social and demographic characteristics, risk factors for environmental lead exposure, and dietary assessment of nutrient intake was collected from all eligible participating mothers.

Infants of participating mothers identified before delivery had umbilical cord blood samples collected at birth (*n* = 520). Children were subsequently assessed for neurocognitive development, and blood lead levels were obtained at 24, 30, 36, 42, and 48 months of age. Data on the neurocognitive test performance are presented elsewhere (Gomaa et al. 2002). The present analysis is limited to data from 422 infants who had archived umbilical cord blood available and were successfully genotyped. After genotyping, the full data set was anonymized to protect the confidentiality of study subjects and to conform with current institutional review board policies. A total of 341 subjects had at least one follow-up blood lead level available and data available on covariates of interest for inclusion in the longitudinal analyses.

### Blood measurements

Blood lead measurements were performed using graphite furnace atomic absorption spectrophotometry (model 3000; Perkin-Elmer, Wellesley, MA, USA) at the ABC Hospital Trace Metal Laboratory in Mexico City according to a technique described by Miller et al. (1987). The laboratory participates in the CDC blood lead proficiency testing program administered by the Wisconsin State Laboratory of Hygiene (Madison, WI, USA), which provided external quality control specimens varying from 2 to 88 μg/dL. Our laboratory maintained acceptable precision and accuracy over the study period [correlation = 0.98; mean difference = 0.71 μg/dL; SD = 0.68].

Complete blood count (red blood cells, white blood cells, hematocrit, hemoglobin, mean corpuscular volume) in manually diluted samples of whole blood (Beckman/Coulter CBC-5 Hematology Analyzer; Block Scientific Inc, Holbrook, NY, USA) and serum ferritin (Ferritin RIA Kit, Kodak Clinical Diagnostics, Ltd. Amersham, Bucks, UK) were measured using standard clinical methods at the ABC Hospital.

### HFE and TF genotyping

We extracted high-molecular-weight DNA from white blood cells of archived umbilical cord blood with commercially available PureGene Kits (Gentra Systems, Minneapolis, MN, USA). After DNA quantification, samples were adjusted to TE buffer (containing Tris, a common pH buffer, and EDTA, a chelating agent), partitioned into aliquots, and stored at −80°C. Multiplex polymerase chain reaction (PCR) assays were designed using Sequenom SpectroDESIGNER software (Sequenom, Inc., San Diego, CA) by inputting sequence containing the single nucleotide polymorphism (SNP) site and 100 base pairs of flanking sequence on either side of the SNP. The extension product was then spotted onto a 384-well spectroCHIP (Sequenom, Inc.) before being flown in the MALDI-TOF (matrix-assisted laser desorption ionization–time of flight) mass spectrometer (Sequenom, Inc.).

For this study, we included three SNPs: hemochromatosis (*HFE*) *C282Y* (rs1800562) and *H63D* (rs1799945) [Reference Sequence NM_139011, GenBank (http://www.ncbi.nlm.nih.gov/entrez/viewer.fcgi?db=nuccore&id=91718887)], and transferrin (*TF*) *P570S* (rs1049296) [Reference Sequence NM_001063, GenBank (http://www.ncbi.nlm.nih.gov/entrez/viewer.fcgi?db=nuccore&id=21536430)]. Specifically, the following primers were used in the multiplex assay:

- For *HFE H63D* (rs1799945): forward PCR primer, 5′-ACGTTGGATGTCTACTG-GAAACCCATGGAG-3′; reverse PCR primer, 5′-ACGTTGGATGTTGAAGC-TTTGGGCTACGTG-3′; extension primer 5′-GCTGTTCGTGTTCTATGAT-3′
- For *HFE C282Y* (rs1800562): forward PCR primer, 5′-ACGTTGGATGTACCCCA-GATCACAATGAGG-3′; reverse PCR primer, 5′-ACGTTGGATGTGGATAAC-CTTGGCTGTACC-3′; extension primer 5′-GAAGAGCAGAGATATACGT-3′
- For *TF P570S* (rs1049296): forward PCR primer, 5′-ACGTTGGATGTGAGTTG-CTGTGCCTTGATG-3′; reverse PCR primer, 5′-ACGTTGGATGATCTTTC-CGTGTGACCACAG-3′; extension primer, 5′-CGCATACTCCTCCACAG-3′.

### Statistical analysis

We examined distribution of *HFE* and *TF* alleles and genotypes and tested frequencies using a chi-square statistic to compare observed and expected counts according to principles of Hardy–Weinberg equilibrium. *A priori* the two *HFE* variants (*H63D* and *C282Y*) were combined into a single indicator term (i.e., presence of one or two copies of either gene’s variant allele), and subsequent analyses compared carriers of *HFE* or *TF* variants with wild-type subjects, thus assuming dominant effects. We calculated summary statistics for child characteristics, stratified by genotype. Bivariate associations between children’s blood lead, ferritin, and hemoglobin levels by genotype (wild type vs. carrier) at each time point were compared using Student’s *t*-test.

Blood lead levels followed a log-normal distribution and were log transformed for the analyses; thus, beta coefficients from regression models represent percent change in blood lead. We used multivariate linear regression to model the effect of genotype on blood lead in cross-sectional analyses at each time point, adjusting for maternal blood lead level at delivery (as a measure of prenatal lead exposure) and child’s concurrent anemia status (as a marker of dietary iron intake). Anemia was defined as a hemoglobin concentration < 12.1 g/dL, based on recommendations for children 2 to < 5 years of age living at an altitude of 7,000–7,999 feet above sea level (CDC 1998). We used random-effects models with unstructured covariance to model the association between *HFE* and *TF* genotype and repeated measures of log-transformed blood lead in separate models. These models are flexible with respect to imbalance in the data and, in addition, take into account the correlation between repeated measures on subjects. To explore a possible gene–gene interaction between *HFE* and *TF* genotypes in predicting blood lead, we modeled combined *HFE* plus *TF* joint genotype as a dichotomous variable (any variant present vs. both wild type) and then, to assess allele “dose” effects, as an ordinal variable (both variants present, *TF* variant/*HFE* wild type, *TF* wild type/*HFE* variant, both wild type) with wild type for both variants as the reference group. Separate models were repeated controlling for concurrent hemoglobin and concurrent ferritin to account for potential differences in dietary iron intake. We also constructed two-way interaction terms between *HFE* and *TF* genotype, and between each gene and lead, hemoglobin, and ferritin levels at each time point to explore potential interactions. Finally, we used logistic regression (PROC NLMIXED) to examine the odds of having a blood lead level ≥ 10 μg/dL associated with presence of gene variants. All statistical analyses were performed using SAS software version 9.1.3 (SAS Institute Inc., Cary, NC, USA).

## Results

Four hundred twenty-two children were genotyped, and 17.7%, 3.3%, and 18.9% carried the *HFE H63D*, *HFE C282Y*, and *TF P570S* variants, respectively (Table 1). In addition, 1% of children carried both the *HFE C282Y* and the *TF P570S* variants, and 3% of children carried both the *HFE H63D* and the *TF P570S* variants (Table 2). There were no statistically significant differences in mean gestational age, sex, umbilical cord blood lead levels, or anthropometric measures at birth between carriers and wild-type subjects (Table 3).

Unadjusted mean blood lead levels were consistently higher for carriers of either the *HFE* or *TF* variant compared with wild-type subjects at each age (24, 30, 36, 42, and 48 months), but these differences were non-significant (Table 4). There were no statistically significant differences in hemoglobin or ferritin levels at any age between carriers and wild-type subjects for either gene variant (results not shown). Unadjusted mean blood lead levels of subjects carrying any variant or both variants (combined *HFE* + *TF* genotype) were consistently higher (at any age) than subjects who were wild type, although these differences were also not statistically significant (Table 4).

In cross-sectional analyses, adjusting for maternal blood lead level at delivery and child’s concurrent anemia status, the relationship between genotype (either *HFE* or *TF*) and blood lead level (at any age) was non-significant (data not shown). However, in the longitudinal analysis (using repeated measures of blood lead at 24, 30, 36, 42, and 48 months of age) adjusting for covariates (maternal blood lead level at delivery and child’s concurrent anemia status), the relationship between *HFE* genotype and blood lead was statistically significant (β = 0.11, *p* = 0.04), and the relationship between *TF* genotype and blood lead was borderline significant (β = 0.10, *p* = 0.08) (Table 5). Thus, on average, carriers of either the *HFE* or *TF* variant had blood lead levels that were 11% and 10% higher, respectively, than wild-type subjects.

There was a statistically significant unadjusted gene-by-gene interaction (β_unadj_ = 0.37, *p* = 0.02) between the *HFE* and *TF* genotypes and, when adjusted for maternal blood lead level at delivery and child’s concurrent anemia status, this interaction term was marginally significant (β_adj_ = 0.31, *p* = 0.06) (data not shown). There were no significant interactions between either gene with concurrent hemoglobin or with concurrent ferritin concentration (data not shown).

We then examined the data to explore whether there was an interactive effect of the presence of both *HFE* and *TF* variants on blood lead levels. We first examined the cross-sectional association between having any variant present (those with *HFE* and/or *TF* variant) versus both wild type. The dichotomous combined *HFE* + *TF* genotype was not significantly related with blood lead (at any age) in cross-sectional models (data not shown). Next, we defined a combined joint genotype by grouping subjects into four categories (both variants, either *HFE* and/or *TF* variant, both wild type) to examine the dose effect for presence of gene variant(s). This specification did not reveal any statistically significant associations with blood lead (at any age) in cross-sectional models (data not shown).

In longitudinal models, the result of having any variant present (β = 0.08, *p* = 0.07) was similar to having either *HFE* (β = 0.11, *p* = 0.04) or *TF* (β = 0.10, *p* = 0.08) variant compared with subjects who were wild type for both variants (Table 5). When examining the variant dose effect, compared with subjects who were wild type for both variants, subjects who carried both *HFE* + *TF* variants had higher blood lead levels (β = 0.41, *p* = 0.006) than subjects who carried either *TF* variant/*HFE* wild type (β = 0.04, *p* = 0.5) or *HFE* variant/*TF* wild type (β = 0.06, *p* = 0.3) (Table 5). Therefore, subjects carrying both variants had blood lead 50% higher than subjects who were wild type for both *HFE* and *TF*. These results were unchanged when adding concurrent ferritin or concurrent hemoglobin (in place of anemia status) as covariates in our models (data not shown).

**Figure 1:**
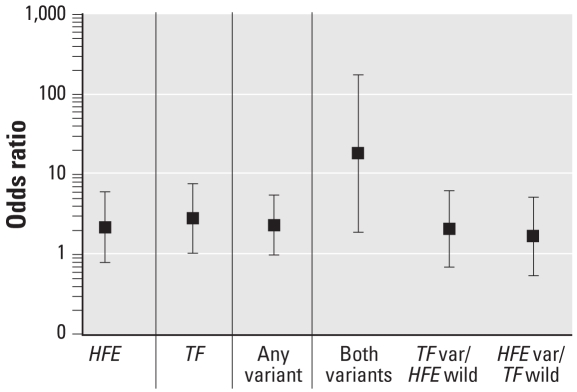
Risk of elevated blood lead levels (≥ 10 μg/dL) by presence of variant allele. OR (and 95% CI) from nonlinear mixed-effects models (adjusting for maternal blood lead level at delivery and child’s concurrent anemia status; reference group = wild type; *n* = 708 observations used; *n* = 340 subjects; maximum number of observations per subject = 3). Vertical lines delineate four separate models. var, variant.

Figure 1 shows the risk of elevated blood lead levels (≥ 10 μg/dL) by presence of variant allele(s). Those subjects with either the *HFE* or *TF* (any) variant present had significantly higher odds of having a blood lead greater than or equal to 10 μg/dL [odds ratio (OR) = 2.3; 95% confidence interval (CI), 1.0–5.5] compared with those who were wild type. Those subjects with both variants present had significantly higher odds of having a blood lead ≥ 10 μg/dL (OR = 18.3; 95% CI, 1.9–177.1) compared with those wild type for both *HFE* and *TF*, though the wide CIs are attributed to the fact that < 5% of our study population had both variants present.

## Discussion

In our current study of Mexican children, carriers of the *HFE* variant genotype had blood lead levels 11% higher than wild-type subjects. Furthermore, carriers of both *HFE* and *TF* variant alleles had 50% higher blood lead levels compared with wild-type subjects in models comparing the joint effect of combined *HFE* + *TF* genotype on blood lead levels. Those subjects with both gene variants present also had significantly higher odds of having a blood lead level ≥ 10 μg/dL. Our results suggest that genes affecting iron metabolism also affect lead metabolism, and this impact may be magnified by the presence of multiple genotypic variant alleles.

This is the first study to examine the association between iron metabolism genes and lead exposure in children. There are at least three previous reports in adults or studies of mixed ages. Barton et al. (1994) found higher blood lead levels in subjects (children to young/middle-aged adults) with HH compared with normal controls, although they used family history and clinical data to determine case status rather than genotype and therefore some subjects in the control group were likely *HFE* variant carriers. In another study, Åkesson et al. (2000) found no difference in blood lead levels between adult subjects (average age, 55.5 years) with hemochromatosis and controls, but found increased blood lead levels in HH patients who had increasing number of years of treatment by phlebotomy, suggesting an up-regulation of absorption (due to lowered iron stores) with a subsequent increase in lead absorption.

In a previous population-based cohort study (Wright et al. 2004), we found lower blood/bone lead levels in hemochromatosis variant (*H63D* or *C282Y*) carriers in a group of elderly men. These results contrast particularly with those of Barton et al. (1994). We hypothesized that the results of these three studies may have differed partly because of variations in body iron stores, which correlate with age and sex. Because of menstrual losses, women tend to have lower body iron stores than men, which would up-regulate iron/lead absorption (Hallberg and Rossander-Hulten 1991). In addition, because no mechanisms for iron excretion exist, in the absence of chronic bleeding disorders, iron stores tend to increase with age. We postulated that the *HFE* gene effect on blood lead level may vary by age because of these differences in iron stores/needs that correlate with age/sex, and that in a younger population with higher body iron needs, the effect of *HFE* variants may be to increase lead absorption among variant carriers. Conversely, in an older population of elderly males, the *HFE* variant effect may be attributable to down-regulation of iron and lead absorption, because iron stores in men are higher than in women. Our current study supports this hypothesis; in a population of young children with high body-iron needs, we found higher blood lead levels among *HFE* variant carriers. This study is among the first to present evidence that gene environment interactions may vary by life stage.

In addition, our current study explored the interactive effect of *HFE* + *TF* combined genotype on blood lead levels in children. Our results suggested that iron metabolism and body lead burden are affected more profoundly by the joint presence of genotypic variant alleles in both *HFE* and *TF*. Given the relatively small sample size to detect gene–gene or gene–environment interactions, we used longitudinal random–effects models, because these models are flexible with respect to imbalance in the data. We were able to include subjects with incomplete data to increase the power of the study to detect these effects.

There are several limitations to our study. Our sample was restricted to a homogeneous sample of Mexican children with a lower prevalence of the *C282Y* variant (heterozygotes, 3.3%) than people from Europe (9.2%) and the Americas (9.0%), but a higher prevalence than people from Africa/Middle East (0.2%), the Indian subcontinent (0.5%), Asia (0%), and Australia (0%) (Hanson et al. 2001). Therefore, because of the wide variability in prevalence rates of the *C282Y* variant (heterozygotes), it will be important to replicate this study in different populations. The potential for misclassification should also be considered, because there may be other polymorphisms in the *HFE* or *TF* genes or in a proximal gene that is in tight linkage disequilibrium with these genes that could account for our findings. Residual confounding is always a concern in observational studies. We chose covariates for this analysis based on biological plausibility as confounders (i.e., factors likely to be associated with both *HFE* genotype and blood lead levels). Among common predictors of blood lead, few are likely associated with *HFE* or *TF* genotype. For example, *HFE* and *TF* are not X-linked; therefore, sex should not be a confounder because it is not associated with *HFE* or *TF* genotype, and any sex-related differences due to menstruation are not yet an issue in a pre-pubescent population. Genotype should also be independent of environmental lead levels. Iron status is plausibly related to blood lead and *HFE* genotype (Wright et al. 2004), but should theoretically be a modifier of the association of *HFE* with blood lead rather than a confounder. Our results did not demonstrate evidence of effect modification by serum ferritin or hemoglobin. Race and socioeconomic status were accounted for in our study design, because we enrolled Mexican children from hospitals that serve a relatively homogeneous group of low-to-moderate income families. Therefore, to account for potential confounding, we controlled for maternal blood lead level at delivery (to account for differences in prenatal lead exposure) as well as child’s concurrent anemia status (to account for differences in dietary iron intake).

With respect to the gene-by-gene interaction findings, a limitation of this study is that carriers of both variants represent < 5% of our study population, and even though the result is statistically significant, this could be a chance finding. However, there is biological plausibility to the relationship between *HFE* and *TF*, and ours is not the first study to find synergy between these two variant alleles. The *HFE* gene product regulates the binding of transferrin to transferrin receptors, which in turn regulates the transfer of iron (and potentially other transferrin-bound metals) across cell membranes (Roy et al. 1999; Salter-Cid et al. 1999). Furthermore, lead has been shown to bind transferrin, down-regulating transferrin gene expression (Adrian et al. 1993). Further evidence of this epistatic relationship is seen in clinical studies. Robson et al. (2004) found that carriers of the C2 variant of the *TF* gene and the *C282Y* allele of the *HFE* gene were at five times greater risk for Alzheimer disease compared with all others, whereas neither allele alone had any effect on risk for Alzheimer disease. Although outside the scope of this study, Beckman et al. (1999) examined the association between genetic variants of iron metabolism (*HFE*, *TF*, and the two combined) and risk for multiple myeloma, breast cancer, and colorectal cancer. In their study, the *HFE* and *TF* genotypes tested separately were not associated with any of these neoplastic disorders, but there was a significant difference between patients and controls with respect to the two genotypes combined.

*HFE* genotype is associated with higher blood lead levels in Mexican children over time. Our results also suggest an interaction between *HFE* and *TF* genotype in predicting higher blood lead in young children. These results differ from reports in elderly adults in which *HFE* variants predicted lower blood lead levels, demonstrating that genetic effects differ by life stage.

In conclusion, iron deficiency has been associated with increases in absorption and deposition of lead (Barton et al. 1978); however, the relationship between iron and lead is complex and not completely understood. There is no evidence that iron supplements themselves change lead levels after lead exposure. In a randomized control trial controlling for initial blood lead level, Mexican children who were administered iron (or iron plus zinc) did not have lower blood lead concentrations than the placebo group. Iron supplementation of these lead-exposed children significantly improved iron status but did not reduce blood lead levels (Rosado et al. 2006). Based on previous research, it has been recommended that iron supplementation should be prescribed only to iron-deficient children, regardless of blood lead levels, and not as a treatment for lead poisoning in children (Wright 1999). Thus, children with genetic variants affecting iron metabolism may present a unique challenge: They may be more susceptible to low environmental levels of lead exposure because of increased lead absorption and may also be resistant to interventions such as iron supplementation. Another study evaluating the association between blood lead concentration and a vitamin D receptor (VDR) gene polymorphism found the VDR-*Fok1* variant to be an effect modifier of the relationship of floor dust lead exposure and blood lead concentration (Haynes et al. 2003). In combination, these results highlight that some children may be more susceptible to lead exposure or absorption, which emphasizes the importance of further reducing lead exposure to protect the most at-risk children around the world. In addition, these children might represent a vulnerable population that may benefit from promising new interventions such as environmental enrichment, as defined by a combination of “complex inanimate objects and social stimulation” (Guilarte et al. 2003; van Praag et al. 2000). Guilarte and colleagues exposed rats pre- and postnatally to lead, and then randomized them to an enriched environment (cage with a greater space allowance per rat, more varied toys and fixed objects, and increased human handling) versus a standard laboratory cage with minimal handling. The rats reared in the enriched environment showed improvement in spatial learning performance, compared with rats reared in the standard laboratory cage, suggesting the enriched environment mitigated some of the neurotoxic effects of lead exposure (Guilarte et al. 2003). These results are particularly applicable to children, as risk factors for increased lead exposure in children include low socioeconomic conditions such as poor housing and decreased maternal education. Therefore, programs aimed at defining and providing environmental enrichment for children at increased risk of lead exposure might not only reduce risk but also mitigate the neurotoxic effects of exposure.

## Figures and Tables

**Table 1:** Genotype frequencies of children born in Mexico City, 1994–1995.

Genotype	No. (%)
*HFE C282Y*^a^	
*C282Y* homozygous wild type (CC)	408 (96.7)
*C282Y* heterozygous (CY)	14 (3.3)
*C282Y* homozygous variant (YY)	0 (0)
*HFE H63D*^b^	
*H63D* homozygous wild type (HH)	345 (82.3)
*H63D* heterozygous (HD)	70 (16.7)
*H63D* homozygous variant (DD)	4 (1.0)
*TF P570S*^c^	
*P570S* homozygous wild type (CC)	339 (81.1)
*P570S* heterozygous (CT)	77 (18.4)
*P570S* homozygous variant (TT)	2 (0.5)

a Eleven children were missing genotype data; results in Hardy–Weinberg equilibrium: χ^2^ = 0.12, *p* = 0.73.

b Fourteen children were missing genotype data; results in Hardy–Weinberg equilibrium: χ^2^ = 0.05, *p* = 0.83.

c Fifteen children were missing genotype data; results in Hardy–Weinberg equilibrium: χ^2^ = 1.16, *p* = 0.28.

**Table 2:** Combined genotype frequencies^a^ of children born in Mexico City, 1994–1995.

	*TF* genotype	
	Wild (CC) No. (%)	Variant (CT or TT) No. (%)
*HFE* genotype		
Wild (CC, HH)	269 (65)	62 (15)
Variant (CY, YY, HD, DD)	65 (16)	16 (4)

a Twenty-one children were missing data on combined *HFE* + *TF* genotype.

**Table 3:** Baseline characteristics of study population by *HFE* genotype and *TF* genotype.

			*HFE* genotype				*TF* genotype			
	All		Wild type		Variant		Wild type		Variant	
Characteristic	No.^a^	Mean ± SD	No.	Mean ± SD	No.	Mean ± SD	No.	Mean ± SD	No.	Mean ± SD
Gestational age (weeks)	413	39.2 ± 1.5	329	39.2 ± 1.5	84	39.1 ± 1.4	337	39.2 ± 1.5	78	39.0 ± 1.6
Sex (% female)	414	44	330	46	84	36	338	43	78	54
Cord blood lead (μg/dL)	364	6.6 ± 3.7	289	6.6 ± 3.5	75	6.9 ± 4.2	296	6.7 ± 3.7	69	6.5 ± 3.8
Birth weight (g)	415	3,149 ± 409	331	3,164 ± 402	84	3,087 ± 433	338	3,147 ± 419	79	3,139 ± 369
Birth length (cm)	411	50.5 ± 2.3	327	50.4 ± 2.3	84	50.5 ± 2.1	335	50.5 ± 2.3	78	50.2 ± 2.1
Head circumference (cm)	399	33.9 ± 1.5	315	34.0 ± 1.5	84	33.7 ± 1.5	324	33.9 ± 1.5	77	33.9 ± 1.5

a Because of missing data, numbers do not equal 422.

**Table 4:** Children’s blood lead levels (micrograms per deciliter) by age and by *HFE, TF,* and combined genotype (at each age).

			*HFE* genotype				*TF* genotype			
	All		Wild type		Variant		Wild type		Variant	
Age (months)	No.	Mean ± SD	No.	Mean ± SD	No.	Mean ± SD	No.	Mean ± SD	No.	Mean ± SD
24	283	8.2 ± 4.4	231	8.0 ±4.3	52	9.1 ± 4.6	227	8.0 ±4.4	55	8.8 ± 4.4
30	167	8.4 ± 3.8	140	8.3 ±3.9	27	9.2 ± 3.7	134	8.4 ±3.9	34	9.3 ± 4.4
36	206	8.4 ± 5.2	164	8.2 ±5.4	42	8.9 ± 4.1	161	8.3 ±5.0	44	8.8 ± 5.9
42	215	8.5 ± 6.2	171	8.4 ±6.6	44	8.7 ± 4.4	169	8.4 ±6.6	45	9.0 ± 4.2
48	227	8.2 ± 3.8	183	8.0 ±3.7	44	8.9 ± 4.2	179	8.1 ±3.7	50	8.6 ± 4.1
	Combined genotype									
					Variant dose effect					
	No variant Both wild type^a^		Any variant *HFE* and/or *TF* variant		*HFE* variant/*TF* wild		*TF* variant/*HFE* wild		Both variants present	
Age (months)	No.	Mean ± SD	No.	Mean ± SD	No.	Mean ± SD	No.	Mean ± SD	No.	Mean ± SD

24	185	8.0 ± 4.5	94	8.5 ±4.2	40	8.2 ± 4.0	45	7.9 ±3.5	9	13.0 ± 6.0
30	110	8.2 ± 3.9	56	8.9 ±4.2	23	9.0 ± 3.4	29	8.7 ±3.8	4	10.6 ± 5.3
36	125	7.9 ± 3.8	77	8.6 ±4.9	34	8.4 ± 3.5	37	8.2 ±5.7	6	11.6 ± 6.7
42	132	8.0 ± 4.3	79	8.5 ±3.9	35	8.1 ± 3.6	37	8.3 ±3.5	7	12.0 ± 6.4
48	141	8.0 ± 3.8	85	8.5 ±3.9	36	8.5 ± 3.6	41	8.0 ±3.2	8	11.0 ± 6.1

a Both wild type is the reference group for any variant and variant dose effect classifications.

**Table 5:** Longitudinal associations^a^ between genotype and log-transformed blood lead levels.

Variable	No.	β	SE	*p*-Value
*HFE* variant present^b^	341	0.11	0.05	0.04
*TF* variant present^b^	341	0.10	0.06	0.08
Any variant present^b^	337	0.08	0.04	0.07
Both variants^c^	337	0.41	0.15	0.006
*TF* variant/*HFE* wild type		0.04	0.06	0.5
*HFE* variant/*TF* wild type		0.06	0.05	0.3

Shading indicates four separate models.

a Adjusted for maternal blood lead level at delivery and child’s concurrent anemia status.

b Reference group is wild-type genotype.

c Reference group is wild type for both genotypes.
